# Suicidal ideation after stroke: why standardized screening is essential for community-based prediction models

**DOI:** 10.3389/fneur.2026.1867538

**Published:** 2026-06-11

**Authors:** Polona Rus Prelog, Matija Zupan, Senta Frol

**Affiliations:** 1 Centre for Clinical Psychiatry, University Psychiatric Clinic Ljubljana, Ljubljana, Slovenia; 2 Faculty of Medicine, University of Ljubljana, Ljubljana, Slovenia; 3 Department of Vascular Neurology, University Medical Centre Ljubljana, Ljubljana, Slovenia

**Keywords:** aphasia, depression, PHQ-9, post-stroke suicidality, prediction models, primary care, standardized assessment, stroke survivors

## 1 Introduction

A substantial body of epidemiological work indicates that stroke confers an increased risk of suicidal ideation, suicide attempts, and suicide deaths compared with the general population (1–5). Suicide is now increasingly recognized as a significant long-term complication of stroke, with large cohort studies and meta-analyses showing that stroke survivors have roughly a 1.7- to almost three-fold higher risk of suicide than people without stroke (3, 5). Population-based data suggest that about 3–4 of every 1,000 stroke survivors die by suicide within the first 5 years, with the highest absolute rates in younger patients and those with shorter initial hospital stays (6–8). Beyond deaths, around one in eight stroke survivors report suicidal ideation, and pooled estimates place the prevalence of suicidal thoughts at about 11%−12% (7), with somewhat higher rates reported in Asian cohorts (9).

At the same time, registry-based and administrative datasets including hundreds of thousands to millions of patients now allow refined estimation of suicide attempts and suicidal behaviors after stroke, with adjusted hazard ratios for suicide attempt around 2.0–2.2 compared with non-stroke controls (5, 10). These data highlight not only traditional clinical risk factors such as post-stroke depression, but also socioeconomic and contextual determinants, including low income, low urbanization, and manual occupations, which are particularly relevant for planning community-level prevention (9–12). Building on this epidemiological foundation, recent work has begun to develop prediction models for post-stroke suicidal ideation that combine routinely collected clinical, functional, and psychological measures with modern analytic approaches, including machine learning, to stratify risk in subacute and chronic stroke survivors (1, 13, 14).

Against this backdrop, there is a growing opportunity—and responsibility—to move from predominantly hospital-based, short-term screening toward community-based prediction models that can identify stroke survivors at elevated risk of suicidality across the continuum of care. By community-based prediction models we refer to tools that use routinely collected information from primary care, rehabilitation services, and population surveys to estimate an individual stroke survivor's risk of suicidality during longer-term follow-up, outside the acute hospital setting.

Community-based prediction models differ from in-hospital screening tools along three main dimensions: data sources, time frame, and deployment. In-hospital screening usually relies on single-time-point questionnaires or clinical interviews during admission or early specialist follow-up, often triggered by visible distress, whereas community-based models integrate repeated symptom scores, disability and comorbidity measures, medication use, and social variables (for example, living situation or employment status) that accumulate in primary-care and rehabilitation records over time. They are designed to estimate future or persistent risk over extended follow-up periods rather than simply detecting current symptoms, and are typically implemented within electronic health records or registry infrastructures as automated risk calculators that update at predefined time points (such as discharge, 3–6 months, and 12 months), and generate prompts for structured review, safety planning, or referral according to local pathways. In this sense, community-based models function as adjunctive decision-support tools that can systematically flag at-risk stroke survivors in less supervised environments, complementing but not replacing in-person clinical assessment (1, 13). Such tools, however, can only reach their potential if they are embedded within standardized approaches to suicidality assessment, and if their limitations, ethical implications, and implementation challenges are explicitly acknowledged. In this Opinion, we argue that further gains in preventing suicide after stroke will depend less on ever more complex algorithms and more on the systematic use of harmonized, stroke-appropriate suicidality measures embedded in community-based prediction models and care pathways.

## 2 Discussion

Despite clear epidemiological evidence that suicidality is a relatively frequent and clinically important complication of stroke (2–5), it is not systematically assessed in many neurology, rehabilitation, or primary-care follow-up pathways and is often explored only when depressive symptoms are overt or specialist psychiatric input is available.

One major obstacle to more consistent practice is the lack of standardization in suicidality measurement in stroke research. Existing studies have employed a wide range of instruments, from single items within generic depression scales [e.g., Patient Health Questionnaire-9 (PHQ-9) item 9 or analogous items in Hospital Anxiety and Depression Scale (HADS) and Center for Epidemiologic Studies Depression Scale (CES-D)] to dedicated suicidality questionnaires and non-standardized clinical ratings, with differing time frames and thresholds for “caseness” (2, 4, 6, 8). Furthermore, relatively few instruments have been formally validated in stroke populations, and even fewer have been adapted for survivors with aphasia or significant cognitive impairment, who may be at least as vulnerable to psychological distress as verbally fluent patients (2, 4, 11). This methodological heterogeneity limits comparability across studies, complicates synthesis of prevalence and risk-factor estimates, and constrains the development of robust and transportable prediction models.

Much of the existing work on suicidality after stroke has centered on brief, hospital-based screening in the acute phase or on the use of generic suicide-risk tools that were not specifically developed for stroke populations. By contrast, this commentary highlights the need for stroke-specific, community-oriented prediction models that are grounded in harmonized, validated suicidality measures and applicable across longer-term follow-up. Current post-stroke follow-up recommendations seldom provide detailed guidance on routine suicidality screening despite robust epidemiological evidence of increased risk, and the strategies outlined here are intended to help address this gap in practice.

Within this context, community-oriented prediction models for depression and suicidal ideation after stroke have begun to use brief instruments such as the PHQ-9 alongside routinely collected sociodemographic, lifestyle, and medical variables from large population surveys and clinical datasets (1, 6, 10, 12–14). Such models often achieve reasonably good discrimination (with AUC values in the mid-0.70s) and acceptable calibration, in line with other post-stroke mental health prediction work, suggesting that clinically interpretable risk stratification may be feasible using information already available in primary care and community settings. For example, a primary-care registry model that updates predicted risk at discharge, 3–6 months, and 12 months using PHQ-9 scores, functional disability, and key social variables (such as living alone or work incapacity) could automatically flag high-risk stroke survivors for structured clinical review and, where indicated, referral to mental health services.

The relatively high weight typically assigned to variables such as marital status, work incapacity, and chronic somatic illness underscores the central role of social isolation, disability, pain, and multimorbidity in post-stroke suicidality, as highlighted by meta-analyses and cohort studies (8, 10). Importantly, most authors emphasize that these predictors should not be interpreted as directly causal—particularly in the case of cardiovascular risk factors and psychotropic medication use—which is methodologically sound and helps guard against simplistic causal inferences in clinical practice.

At the same time, recent models exemplify structural constraints of current suicidality research in stroke. Reliance on a single PHQ-9 item to capture suicidal ideation is consistent with primary-care practice and with evidence linking item 9 to subsequent suicidal behavior, but it conflates passive death wishes with more active self-harm thoughts and has not been comprehensively validated in stroke cohorts, especially among individuals with aphasia or cognitive impairment (8, 9). Moreover, large survey infrastructures and self-report-based cohorts necessarily under-represent survivors who are unable to complete self-report questionnaires, meaning that some of the most clinically vulnerable patients are largely absent from model derivation and validation (2, 4).

These limitations are consistent with broader evidence that PHQ-9 item 9 alone misses a substantial proportion of individuals at risk of suicidal behavior and is insufficient as a stand-alone assessment of suicide risk, even though higher scores are robustly associated with subsequent attempts and deaths (15, 16). This combination of imperfect sensitivity with high feasibility helps explain its widespread use in primary care and post-stroke depression screening, but also underlines that it should function as an initial flag requiring more detailed follow-up rather than a definitive measure of suicidality and that complementary work on stroke-specific suicidality measures and broader validation across settings is needed.

These observations suggest that the primary value of such work lies not only in the specific techniques produced, but in what they signal about the next steps needed to integrate suicidality assessment into post-stroke care. On this basis, several implications for practice and research can be outlined.

First, suicidality assessment should be incorporated explicitly, systematically and routinely into post-stroke follow-up across disciplines. In many neurology, rehabilitation and primary-care settings, the most immediately implementable option is to embed one or two brief suicidality questions, such as PHQ-9 item 9 supplemented by standardized follow-up questions on intent, planning, and past behavior, within routine depression screening, as this builds on existing workflows and tools that have at least partial empirical support for identifying elevated risk (17, 18). For patients with communication or cognitive impairments, stroke-adapted instruments and structured collateral information from caregivers should be developed and piloted in parallel; in this sense, reliance on PHQ-9 item 9 can be seen as a transitional strategy while more stroke-specific suicidality measures are validated and progressively integrated into practice.

Second, there is a clear research need to converge on a limited set of suicidality measures that are psychometrically robust and clinically meaningful in stroke populations. Instruments should be validated in diverse samples, including those with aphasia and cognitive impairment, with clearly specified time frames and cut-offs and guidance for interpretation in different care settings. In practical terms, harmonization would mean selecting a small number of core suicidality items or scales with clearly specified time frames (for example, past 2 weeks vs. past month), response options, and cut-points that are used consistently across stroke studies and follow-up clinics. These instruments should be adapted for aphasia and cognitive impairment, with parallel self-report and informant-report versions where appropriate, and accompanied by structured guidance on how to respond to different levels of risk (including when to escalate to urgent assessment). Agreement on a core suicidality item set for stroke research, analogous to core outcome sets in other fields, would enhance comparability of prevalence estimates, enable pooled analyses and external validation of prediction models, and simplify integration into electronic health records and registries (15, 18). Candidates might include a stroke-validated adaptation of PHQ-9 item 9 or brief items from the Columbia-Suicide Severity Rating Scale, alongside structured follow-up questions on intent and prior attempts, but these require systematic testing in stroke populations (16, 18).

Third, prediction models are best conceptualized as adjunctive risk-stratification tools within defined care pathways, rather than as stand-alone decision instruments (4). Their principal role should be to identify individuals who warrant comprehensive clinical assessment of depression and suicidality and, where appropriate, referral to mental health services. Any implementation should occur in settings where clear protocols for follow-up assessment and intervention exist and should be accompanied by training that emphasizes the non-causal interpretation of individual predictors and the limitations of single-item suicidality measures.

Embedding systematic suicidality assessment and risk-stratified care into post-stroke follow-up also faces important implementation barriers. At the clinic and provider level, time pressure, competing priorities, limited training in suicide risk assessment, and uncertainty about how to respond to positive screens are recurrent obstacles reported in primary-care implementation studies. At the patient and family level, stigma, discomfort discussing suicide, concerns about the consequences of disclosure, and cultural differences in how suicidality is expressed can affect both the acceptability and accuracy of screening. Addressing these barriers will require not only brief, stroke-appropriate instruments, but also clear local protocols for managing positive results (including same-day safety planning and referral options), staff training that emphasizes culturally responsive communication, and organizational support to integrate screening into existing workflows (18, 19).

Finally, prospective, geographically diverse cohort studies using harmonized suicidality definitions are required to evaluate whether systematic screening and risk-stratified care actually improve outcomes. Such studies should examine trajectories of suicidal ideation and behavior after stroke, and test whether integrating standardized suicidality assessment and risk-stratification tools into routine practice leads to reductions in suicidal ideation, attempts and deaths, and to improvements in patient-centered measures such as quality of life and functional recovery. More broadly, advancing suicidality assessment after stroke will require alignment between epidemiology, instrument development, prediction modeling and implementation science, so that screening becomes both standardized and meaningfully embedded in real-world care pathways.

## 3 Conclusions

Post-stroke suicidality should be treated as a routine target of follow-up, not as an exceptional complication addressed only when patients explicitly disclose suicidal thoughts. Stroke services and primary-care providers ought to incorporate at least one brief, validated suicidality item (for example PHQ-9 item 9 with standardized follow-up questions) into scheduled reviews at key time points such as discharge, 3–6 months, and 12 months, with clear local protocols for urgent assessment and referral when risk is identified. Finally, we propose that research programmes prioritize validating suicidality instruments in aphasia and cognitive impairment, agreeing on a small set of core measures, and externally testing community-based risk models in diverse stroke cohorts, so that prediction tools can be implemented as transparent, adjunctive aids within defined care pathways rather than opaque stand-alone algorithms.
